# Anti-HBs levels in children under the age of two years born to HBV carrier mothers after immunoprophylaxis: a multicenter cross-sectional study

**DOI:** 10.1186/s12887-021-02967-8

**Published:** 2021-11-04

**Authors:** Min Jiang, Bo Zhu, Qiang Yao, Haifeng Lou, Xiaohui Zhang

**Affiliations:** 1 Department of Epidemiology and Health Statistics, West China School of Public Health and West China Fourth Hospital, Sichuan University, No. 17 Section 3, Renmin South Road, Chengdu, Sichuan 610041 People’s Republic of China; 2 Department of Medical Laboratory Science, Women’s Hospital, School of Medicine, Zhejiang University, Xueshi Road 1, Hangzhou, Zhejiang 310006 People’s Republic of China; 3 Department of Obstetrics, Women’s Hospital, School of Medicine, Zhejiang University, Xueshi Road 1, Hangzhou, Zhejiang 310006 People’s Republic of China; 4 Department of Women’s Health, Women’s Hospital, School of Medicine, Zhejiang University, Xueshi Road 1, Hangzhou, Zhejiang 310006 People’s Republic of China

**Keywords:** HBV markers, HBV immune response, HBV carrier women, Preterm birth, Low birth weight

## Abstract

**Background:**

Serological testing for the presence of Hepatitis B Virus (HBV) markers and anti-HBs titers in infants born to HBsAg positive women is critically important for estimation in immunisation programme.

**Methods:**

This was a multi-center and cross-sectional study conducted in Zhejiang province, China. Children aged 7 to 24 months born to HBsAg positive women during December 2018 to February 2019, completed additional HBV serological markers screening. We indicated distribution of HBV serological markers and anti-HBs titers in children. Multiple logistic regression model with adjusted odds ratio and 95% confidence interval (OR_*adj*_ and 95% *CI*) was used to explore the factors associated with inadequate immune response (anti-HBs titers< 100 mIU/ml) among children.

**Results:**

A total of 1849 children were included. Overall 25 children tested HBsAg positive, giving HBsAg positive rate of 1.35%(95%*CI*: 0.83-1.88%). 92.00% (23/25) HBsAg positive children were delivered by HBeAg positive mothers. The proportion of protective seroconversion (anti-HBs titers≥10mIU/ml) was 99.29% in all children, and 86.48% children were reported with adequate anti-HBs titers (≥100mIU/ml).We found a significant higher proportions of early antenatal health care (< 13 gestational weeks), and term birth in children with adequate response compared with inadequate response (all *P* < 0.05). Logistic regression showed preterm birth was a negative factor for inadequate anti-HBs titers (OR_adj_ = 1.868,95%CI 1.132-3.085,*P* = 0.015).

**Conclusions:**

Children delivered by HBeAg positive mothers had higher risk of vertical transmission of HBV, despite completion of 3 doses of hepatitis B vaccine and HBIG injection. Inadequate anti-HBs level was significantly associated with preterm birth in HBsAg positive women.

## Introduction

Vertical transmission is the main route for young children to have Hepatitis B Virus (HBV) infection. With the wide coverage of vaccination against HBV and Hepatitis B immune globulin (HBIG) for newborns from HBV-infected women, mother to child transmission (MTCT) of HBV has declined globally [1–4]. In some countries or regions, HBsAg positive rates in children at age five was even less than 1% [3, 5]. In 2016, World Health Organization (WHO) called for ending HBV as a public health threat with the achievement goal of sustainable development (2030), aiming to reduce HBV prevalence under 1% by 2020 and under 0.1% by 2030 among children at the age of five [4, 5]. Nevertheless, there were still gaps to meet the global HBV elimination goal due to the disparity in social-economical development, HBV epidemiology and coverage of prevention MTCT [2–7]. For example, some countries or regions in Africa and Asia still face high endemic of HBV, particularly in women without vaccination [7, 8]. In central and eastern Europe, only 75% countries provide universal HBV screening for pregnant women [6]. Globally, less than 50% countries are available for providing first HBV vaccine dose within 24 h by WHO [2].

HBV vaccination along with HBIG are effective to reduce MTCT [2]. Postvaccination serological testing (PVST) is important for evaluating the immunization effects, although it is not routinely performed in some countries. In some previous studies, no response, protective response, adequate response and high response to vaccine were regarded as anti-HBs titers < 10 mIU/mL, anti-HBs ≥ 10 mIU/mL, anti-HBs ≥100 mIU/mL, and anti-HBs ≥ 1000mIU/mL, respectively [9–11]. Anti-HBs ≥ 10 mIU/mL is widely considered as a seroprotective response to vaccine [9]. Furthermore, some studies preferred anti-HBs titer exceeding 100 mIU/mL as protective effect [11, 12].Anti-HBs titer less than 100mIU/mL was indicated as risk factor for HBV reactivation, and booster vaccination was suggested [11, 13]. A study showed that after completion of HBV immunization course in infancy;5-20% infants encountered vaccination failure or poor response [9–14]. Currently, there are a few studies on infant seroprotective level of anti-HBs over100mIU/mL.

China has the largest burden of HBV infection in the world with 5-6% prevalence and estimated number of HBsAg carriers around 70 million [15]. Chinese government has made great efforts to control HBV. Since 1992, an integrated national immunization programs have included 3 doses of HBV vaccine for infants within 24 h of birth, and subsequently at one and 6 months of birth [1]. Following this, a national strategy of preventing mother to child transmission (PMTCT) of HBV was implemented in China in 2010 [16]. This includes routine HBV screening for pregnant women during their first antenatal health care visit (ANC), and timely vaccine with HBIG for all newborns from HBsAg positive women. PVST is recommend for infants born to HBsAg positive women but not funded by government. Although, China has made great achievement in combating HBV over the past decades, persisting efforts on HBV prevention is still needed considering huge population and geographical variety [15–17]. Zhejiang Province is a relatively developed area in Eastern China with approximately 60 million residents, where the prevalence of HBsAg in people aged 1 to 29 years was 1.05% in 2014 [18]. Since 2016, Chinese Center for Disease Control and Prevention initiated and funded PVST in Zhejiang. One year later, Zhejiang was appointed as a pilot province for the elimination MTCT (EMTCT) of HIV, Syphilis and HBV in China. The coverage, timeliness and completeness of HBV vaccine improved greatly over the past decades, reaching 94-98% in 2017 [19]. To accelerate the progress towards eradication of HBV, we performed the study to verify the HBsAg positive rates, anti-HBs titers and factors associated with adequate immune response among children under two years old, delivered by HBsAg positive women in Zhejiang province. The findings would be of significance for guiding HBV EMTCT.

## Materials and methods

### Study design and subjects

This was a multi-center cross-sectional study. We enrolled subjects in eight urban and rural regions (Ouhai, Pujiang, Ruian,Yuhang, Cixi, Huangyan, Jiaojiang, Ninghai) of Zhejiang province by multi-step sampling according to location, maternal HBsAg positive rate and live births. A total of 2000 singletons aged 7 -24 months and born to HBsAg positive women were recruited during December 2018 to February 2019. A structural questionnaire was used for data collection by medical staff in local women and children’s hospital. We performed a face to face interview with children’s parents or guardians in each local hospital. Information regarding on maternal social-demographic characteristics, ANC, maternal HBV infection status, birth outcomes, HBV immunization and feeding pattern were recorded. All participants were given an informed consent and agreed to participate. The study was approved by Ethnic Committee of Women’s Hospital School of Medicine Zhejiang University.

### EMTCT HBV project

According to Zhejiang provincial HBV EMTCT project, all pregnant women are required to receive HBsAg screening during their first ANC visit and before delivery. HBsAg positive women routinely get further serology testing and HBV DNA tested. Antiviral treatment is recommended to high risk women (a viral load threshold of 2 × 10^5^ IU/ml or HBeAg positive) from 24 gestational weeks. All infants are recommended to receive the first dose of HBV vaccine within 24 h after birth, followed by the second dose at one month and third dose at 6 months of birth. To prevent mother to child transmission, infants born to HBsAg positive women are vaccinated after birth with 100 IU HBIG within 12 h (as early as possible) in delivery hospital, in conjunction with HBV vaccination. We suggest all infants to receive the HBV vaccination impact estimation at least one month later after the third dose, but it is not a mandatory requirement.

### Serological tests

In the study, all serum specimens were tested in the laboratory of Women’s Hospital, School of Medicine Zhejiang University. HBV serological markers presented as Hepatitis B surface antigen (HBsAg), antibody against HBsAg (anti-HBs), Hepatitis B e antigen (HBeAg), antibody against HBeAg (anti-HBe), and antibody against Hepatitis B core antigen (anti-HBc). These five HBV markers were detected by Chemiluminescent Enzyme Immunoassay (CLEIA) with automated immunoassay analyzer (HISCL-5000; Sysmex corporation, Japan). Serum HBsAg and anti-HBs are determined quantitatively while serum HBeAg, anti-HBs, and anti-HBc were determined qualitatively. Specimens with concentrations ≥0.03 IU/mL were considered as positive by HBsAg criteria and ≥ 10mIU/mL as positive on anti-HBs testing.

### Sampling method and statistical analysis

We assumed the HBV MTCT would be about 2%. The calculated average sample size is 169 in each region when we assume 80% power, 95% confidence level, and the precision at 5%. In consideration of the number of regions, the final sample size was 2000 considering 20% possible missing data. PASS 11.0 was used to calculate sample size. We defined adequate immune response as anti-HBs titers ≥100mIU/mL, and others as inadequate.

Distribution of children HBV markers and anti-HBs levels were presented with number and proportion. Chi-square test was used to compare differences between women and children’s adequate and inadequate response characteristics. Multiple logistic regression model with Odds ratio and 95% confidence interval (*OR* and 95% *CI*) was used to explore the risk factors associated with inadequate immune response in children. Significant differences were considered as *P* value < 0.05 with two side effects. All data were double-inputted and checked for consistency using EPI Data software (version 3.02, The EpiData Association).We conducted statistics analysis using SPSS 16.0.

## Results

A total of 1849 children were involved in this study finally. Of these, 23.20% (429) were born to HBeAg positive women who had first HBV screening during pregnancy. Overall, there were 967 boys, 875 girls and 7 unknown gender. The average age of children was 15.30 ± 3.98 months, with a median age of 15.00 months. All children completed three doses of hepatitis B vaccine, including 99.13%(1833) children with 10 μg/dose vaccine and 0.87%(16) without clear information on specific dose of vaccine.94.38% (1745) infants had their first dose of HBV vaccine within 6 h after birth, 2.33%(43) between 6 and 12 h, 2.70% (50) between 13 and 24 h,0.11% (2) beyond24 hours after birth and 0.49% (9) without specific information. Similarly, 95.08% (1758) infants had HBIG within 6 h after birth, 1.94% (36) between 7 and 12 h, 2.60% (48) between 13 and 24 h and 0.38% (7) infants beyond 24 h after birth.

In the study, 1824 children tested negative for HBsAg. 25 children were HBsAg positive, giving overall HBsAg positive rate 1.35% (95% CI 0.83-1.88%). 92.00% (23/25) HBsAg positive children were born to HBeAg positive women. 30.43%(7/23) of these HBeAg positive women received their first ANC beyond 13 gestational weeks. However, only one HBeAg positive woman who delivered HBsAg positive infant had antiviral treatment. In children with negative HBsAg, 2.14% tested both anti-HBc positive and anti-HBe positive, and 0.99% tested only anti-HBc positive.

Anti-HBs titers in HBsAg negative children ranged from 0.13mIU/mL to 8976.11mIU/mL. The seroprotection rate (anti-HBs titers≥10mIU/mL) was 99.29% (1811/1824). The proportion of children with adequate anti-HBs titers(≥100mIU/mL) was 87.34%(1593/1824). Non-response was observed in 0.71%(13/1824) children (Table 1).

**Table 1:** Distribution of HBV markers in children

Subtype		Number	Proportion (%)
HBsAg+		25	1.35
	HBeAg+	23	1.24
	HBeAg +,anti-HBc+	20	1.08
	anti-HBe+,anti-HBc+	0	0
HBsAg-		1824	
	anti-HBc+,anti-HBs - HBeAg-, anti-HBe -	18	0.99
	anti-HBc+, anti-HBe +, anti-HBs -,HBeAg-	39	2.14
	anti-HBs +,anti-HBe +, anti-HBc+, HBeAg-	0	
	anti-HBs ≥ 10mIU/ml	1811	99.29
	anti-HBs ≥ 100mIU/ml	1593	87.34
	10mIU/ml ≤ anti-HBs < 100 mIU/ml	218	11.95
	anti-HBs < 10 mIU/ml	13	0.71

No significant differences were observed in distribution of maternal age, gravidity, parity, employment, maternal HBsAg status maternal abnormal Glutamic-pyruvic Transaminase (ALT) or Glutamic Oxaloacetic Transaminase (AST), delivery mode, boys or girls,low birth weight (LBW) feeding mode and injection time of HBIG between adequate and inadequate immunization groups. Children with adequate response had significant higher proportion of maternal early ANC, and lower proportion of preterm birth than those with anti-HBs titers under 100mIU/mL (Table 2). Multiple logistic regression mode showed only preterm birth (OR_adj_ = 1.868,95%CI 1.132-3.085,*P* = 0.015), adjusted for LBW and ANC was strongly associated with anti-HBs titers under 100 (mIU/mL).

**Table 2:** Comparison between women and children’s adequate and inadequate response characteristics

Variable		Adequate responders		Inadequate responders		χ^2^	*P*
		(*N* = 1593)		(*N* = 231)			
		n	%	n	%		
Maternal age	< 25	93	86.11	15	13.89	2.151	0.542
	25-29	528	88.29	70	11.71		
	30-34	557	85.96	91	14.04		
	≥35	415	88.3	55	11.7		
Gravidity	1	387	84.87	69	15.13	3.988	0.136
	2	489	87.17	72	12.83		
	≥3	711	88.76	90	11.24		
	Missing	6	100	0	0		
Parity	1	10	100	0	0	1.834	0.400
	2	614	87.84	85	12.16		
	≥3	965	86.86	146	13.14		
	Missing	4	100	0	0		
First antenatal care	first trimester	1244	88.04	169	11.96	6.962	**0.031**
	Second trimester	298	86.38	47	13.62		
	Third trimester	51	77.27	15	22.73		
Employment status	Fixed employment	241	88.28	32	11.72	1.022	0.796
	Service	364	87.5	52	12.5		
	Farmer	105	84.68	19	15.32		
	Unemployed	883	87.34	128	12.66		
Maternal HBeAg during first ANC	HBeAg +	352	86.06	57	13.94	0.994	0.319
	HBeAg -	1030	87.96	141	12.04		
	Unknown	211	86.48	33	13.52		
ALT/AST	Normal	1369	87.03	204	12.97	3.439	0.064
	abnormal	109	81.34	25	18.66		
	missing	115	98.29	2	1.71		
Mode of delivery	Vaginal delivery	812	88.17	109	11.83	0.535	0.465
	Cesarean section	758	87.03	113	12.97		
	Missing	23	71.88	9	28.13		
Gender of children	Female	747	86.66	115	13.34	0.692	0.406
	Male	840	87.96	115	12.04		
	Missing	6	85.71	1	14.29		
Low birth weight(< 2500 g)	Yes	40	78.43	11	21.57	3.761	0.052
	No	1553	87.59	220	12.41		
Preterm	Yes	81	79.41	21	20.59	6.147	**0.013**
	No	1512	87.91	208	12.09		
	Missing	13	86.67	2	13.33		
Feeding within 6 months	Breast	683	89.05	84	10.95	3.839	0.147
	Mixed	432	85.54	73	14.46		
	Artificial	474	86.5	74	13.5		
	Missing	4	100	0	0		
HBIG	Within 12 h	1503	87.28	219	12.72	0.079	0.779
	Over 12 h	90	88.24	12	11.76		

Adequate response indicated anti-HBs titers at or over 100 mIU/mL, inadequate response meant anti-HBs titers under 100 mIU/mL

## Discussion

In our study, the overall HBsAg positive rate was 1.35% among children aged 7-24 months. The global estimation of HBV infection prevalence in children at 5 years old in 2016 was1.4% [20]. Our HBsAg positive rate was lower than studies performed in Japan (1.9%), Malaysia (2.6%), and Denmark(2.3%), targeted on infants, young children or adolescents born from HBV carrier mothers [21–23]. In China, HBsAg positive rate ranged from 0.35% in children at the age of 7 months to 12 years in Jiangsu, 4.9 and 1.4% in children of 13-24 months and 7-12 months in 4 northwest provinces, and 0.9% in children aged 7-22 months in Hebei Guangdong Shanxi and Zhejiang [24–26]. We noticed over 90% HBsAg positive children were born to HBeAg positive women. Among HBsAg positive children, HBeAg positive was prevalent. Delay of first ANC in HBeAg positive women might delay treatment, possibly increasing the risk of vertical transmission. In this study, only one HBeAg positive woman who delivered HBsAg positive child received treatment. We also noticed no vertical transmission occurred in the children delivered by women who had effect antiviral treatment, corresponding to previous report [27]. Therefore, antiviral treatment should be given priority in HBeAg positive women.

Combined passive and active immunization greatly contributed to reduction of HBV infection. A meta-analysis covering 26 studies showed people without vaccination had substantially higher HBsAg prevalence than those universally vaccinated [28]. We noted that over 99% of children developed protective anti-HBs (≥10 mIU/mL), and 87.34% children with anti-HBs levels ≥100 mIU/ml. Previous studies showed protective seroconversion rates varied with children’s age and disease. The anti-HBs positivity rate ranged from around 70% to beyond 95% in normal infants or adolescents [10, 12, 14, 29], but only around 50% among school age children with disease, such as autism spectrum disorders orceliac disease [30, 31]. In our study, the proportion of children with anti-HBs titers at or over 100mIU/mL were slightly less than infant in Changchun at age of 7-12 months(96.5%), but far higher than infants at 1 year in Chongqing (67.8%) and Al-Quds University (62.3%) [9, 11, 32]. In theory, antibody levels naturally declined by age, particularly more rapidly in children over 5 years old [10, 11, 32]. It was shown that over 90% children with initial vaccination failure might be revaccinated successfully [10]. A few proportion of children who tested HBsAg negative were anti-HBc positive. This indicated that these children might have occult HBV infection or have high risk of viral reactivation. Therefore, to analyse the HBV markers in serum is helpful for new intervention programme.

Parent HBV status, maternal HBV-DNA, timely and complete intervention, genetic effects and feeding mode were common determines for HBV vaccination response [9, 12, 26, 29, 32–34]. Overall, a genetic effect could explain 70-90% HBV vaccine responses, the remaining were unknown or environment factors [12]. Nevertheless, few studies focused on the risk factors related to anti-HBs titers under 100 mIU/mL. In our study, the negative association between LBW and anti-HBs less than 100mIU/mL was consistent with the study in North China and Xinjiang to some extent (both with adjusted OR = 2.5) [9, 12].LBW or preterm birth were the common reasons for delay in vaccination, which possibly lead to no or inadequate response. Breast feeding did not increase the risk of poor vaccination response in our study as previously reported [9, 33, 34]. In the United States, women with HBV infection are widely encouraged to breast-feed if the infant receives HBV vaccination and Hepatitis B immunoglobulin fully, which is similar to our province [34].

It has been widely evidenced that positive maternal HBeAg infection increases risks of MTCT [25, 35–37]. For HBeAg positive mothers, higher HBV-DNA level was strongly associated with increased risk for poor response to vaccination [9]. Exploration of risks related to maternal viral load in our study was limited due to lack of specific information, notably only 64 women had clear records of HBV DNA testing. The association between high anti-HBs titres and maternal HBeAg status is unclear. As previously suggested, protective effect is significant when infants born to HBeAg positive women were injected 20 μg HBV vaccine within 2 h after birth [9]. Exploration of different immunization projects according to maternal HBV status should be given much consideration.

In this study, we focused on inadequate response to HBV vaccine in children. This provides further helpful information for HBV vaccination and EMTCT, particularly for high risk women and their infants. There were several limitations in our study. Firstly, the study did not involve fathers basic information, which may also indicate source of children’s infection [38, 39]. Secondly, we only recruited local resident children and singletons births, which might bring some selective bias. Thirdly, influences by maternal viral load and treatment was not involved due to limited data.

## Conclusions

Hepatitis B vaccine and HBIG injection would result in protective antibody in over 99% infants. Preterm birth need to be considered for inadequate response to the vaccine. Moreover complete routine infant HBV vaccination, vertical transmission was observed in infants born to HBeAg positive women.

## Data Availability

The datasets generated and/or analysed during the current study are not publicly available but are available from the corresponding author on reasonable request.

## Abbreviations

HBV: Hepatitis B virus
HBIG: Hepatitis B Immunoglobulin
OR: Odds Ratio
ORadj: Adjusted Odds Ratio
CI: Confidence Interval
anti-HBs: anti-HBV Surface Antigen
MTCT: Mother to Child Transmission
HBsAg: Hepatitis B Surface Antigen
WHO: World Health Organization
PVST: Postvaccination serological testing
PMTCT: Preventing mother to child transmission
EMTCT: Elimination MTCT
HBeAg: Hepatitis B e antigen
anti-HBe: Antibody against Hepatitis B e antigen
anti-HBc: Antibody against Hepatitis B core antigen
ANC: Antenatal health care
CLEIA: Chemiluminescent Enzyme Immunoassay
ALT: Glutamic-pyruvic Transaminase
AST: Glutamic Oxaloacetic Transaminase
LBW: Low birth weight
